# Assessment of the Exercise Intensity of Short Stick Exercises in Elderly Individuals

**DOI:** 10.1155/2015/209368

**Published:** 2015-02-03

**Authors:** Shigeki Kurasawa, Katsushi Yokoi, Nobuyuki Miyai, Kouichi Yoshimasu, Shigeki Takemura, Kazuhisa Miyashita

**Affiliations:** ^1^Department of Rehabilitation Sciences, Kansai University of Welfare Sciences, 3-11-1 Asahigaoka, Kashiwara 582-0026, Japan; ^2^School of Health and Nursing Science, Wakayama Medical University, 580 Mikazura, Wakayama 641-0011, Japan; ^3^Department of Hygiene, School of Medicine, Wakayama Medical University, 811-1 Kimiidera, Wakayama 641-8509, Japan

## Abstract

The present study was to obtain basic data for applying the short stick exercises to frail elderly individuals. A total of 20 individuals aged ≥60 years (10 men, and 10 women) with independence in activities of daily living participated in a short stick exercise program. During the exercise program, the time required and the number of times the short stick was dropped were investigated. The exercise intensity was also evaluated based on expired gas and heart rate measurements. The mean exercise intensity of the short stick exercises was 1.9 ± 0.3 metabolic equivalents (METs), equivalent to talking while standing or walking indoors. Compared to the early elderly (those aged 60 to 74 years), the late elderly (those aged ≥75 years) had a significantly higher number of stick drops and significantly lower increase in heart rate from resting to the warming-up exercise. The short stick exercises had a low exercise intensity and can be applicable to exercise interventions of the frail elderly individuals. However, in the case of the late elderly, the high frequency of short stick drops and the change in heart rate during warming up must be considered.

## 1. Introduction

Meta-analyses have demonstrated a positive link between the amount of physical activity and longevity [1, 2]. Studies targeting elderly individuals have shown that limited physical activity acts as a predictive factor of impairment of activities of daily living (ADL) [3, 4]. Exercise intervention for the elderly has also been proven to be effective in preventing falls and dementia [5, 6]. In regard to aerobic exercises, the American College of Sports Medicine recommends that elderly individuals engage in moderate-intensity exercises (3 to 6 metabolic equivalents (METs)) for a minimum of 30 to 60 minutes per day, at least 5 times a week or more [7]. However, the elderly are more prone to developing various illnesses such as heart disease and arthritis with advancing age, and many individuals are unable to engage in moderate-intensity exercises as noted above. We contrived the short stick exercises (SSEs) which involves the throwing and catching of a short stick while sitting in a chair to accustom the elderly to postural imbalance, thus preventing their falling and stumbling [8]. The SSEs could be a fall prevention exercise focused on the modifiable risk factors of physical functions for falls. The SSEs consisted of six sessions. Session 1 was for warming up, and sessions 2 through 6 included throwing, catching, keeping, flipping, and dropping a short stick. Since these exercises use a “stick” made of rolled newspaper, they are economical, and, characteristically, they are less likely to be limited by time or place. Furthermore, since these exercises can be done while sitting down, the individuals can safely shift their weight and engage in physical activities such as stretching. We have already reported the effectiveness of the SSEs as a fall prevention measure in a cluster randomized trial [9]. As secondary effects of engaging in the SSEs, in our previous study, we observed improvements in 30-second chair stand (CS-30) test, functional reach test, falling stick reaction test, timed up and go (TUG) test, one leg balance (OLB) time, seated forward bend score, and subjective perception of health. These findings demonstrate that SSEs may contribute to not only preventing the elderly from falling but also improving basic physical fitness. When the subjective intensity of the SSEs was assessed among healthy elderly individuals, the results showed that the SSEs are fairly light to somewhat hard intensity exercise [10] according to Borg's rating of perceived exertion scale [11]. However, in order to adapt the SSEs for individuals that are at a higher health risk than late elderly individuals, the intensity of physical activity and cardiorespiratory responses of the SSEs must be examined in detail. The objective of this study was to determine the intensity of physical activity and cardiorespiratory responses of the SSEs when conducted by elderly individuals and to collect basic data necessary for adapting the SSEs to meet the needs of frail elderly individuals.

## 2. Materials and Methods

### 2.1. Subjects

The participants of this study consisted of local residents aged 60 years and older with independent ADL who did not have dementia and were not under physical activity restrictions from their physicians. Study subjects were recruited using university campus bulletin boards and by calling on local residents. A total of 20 individuals (10 men and 10 women) who had no previous experience of the SSEs participated in the study. Prior to conducting the study, all subjects were given an oral and written explanation of the study, and written informed consent was obtained. This study was approved by the research ethics committee of the Kansai University of Welfare Sciences.

### 2.2. Short Stick Exercises

The exercise routine consisted of the following 6 sessions: Session 1, “warming up”; Session 2, “throwing and catching stick with one hand”; Session 3, “throwing and catching stick with both hands”; Session 4, “keeping balance of stick”; Session 5, “flipping stick over”; and Session 6, “dropping the stick.” Details of each exercise can be found in Table 1. Since the SSEs were designed so that each individual can perform the fixed exercise routine at his or her own pace, the time and speed required to perform each exercise varied among different individuals.

### 2.3. Measurement

Subjects were asked to refrain from engaging in any strenuous exercises or consuming alcohol in the evening before the test. Caffeine intake and smoking were also prohibited on the day of the test. Subjects were asked to refrain from eating 2 hours prior to the start of their test. The study period was between January 23, 2013, and March 29, 2013, from 09:00 a.m. to noon. All tests were conducted in a test room kept at a temperature between 22°C and 24°C. No one was allowed in or out of the test room during the SSEs test. After the subjects were guided into the test room, they were asked to sit still for 10 minutes before their blood pressure was measured. A total of 3 measurements were taken with 30- to 60-second intervals between them. The resting blood pressure was obtained by calculating the average of the second and third readings. Next, a portable expired gas analyzer (AT-1100, Anima Corp., Chofu City, Tokyo, Japan) was placed over the subject. The subject was asked to sit quietly until the MET readings and heart rate stabilized. Data were collected for 3 minutes under resting conditions, and an average value was obtained. This value was considered to be 1 MET. After measuring expired gas at resting conditions, the subject began the SSEs led by the SSE creator. In order to ensure the subject's safety during measurement, one researcher closely monitored and accompanied the subject at all times during the exercises to prevent falling. To prepare for unforeseen circumstances, test measurements were conducted in close cooperation with the adjacent medical facility. The following items were measured as expired gas analysis indices: oxygen uptake (*V*_O_2__), oxygen pulse (O_2_ pulse), minute ventilation (*V*_*E*_), and metabolic equivalents (METs). Heart rate (HR) was also measured. The expired gas was recorded based on the breath-by-breath system, and, along with the HR, it was measured starting from the resting period to the end of the SSEs. Using the new rating of perceived exertion (RPE) as an indicator of subjective intensity of physical activity, each subject's evaluation was obtained following the termination of measurements with the expired gas analyzer. In order to study the characteristics of the SSEs, total time required to complete the SSEs and the number of unintentional stick droppings on the floor were also measured. Furthermore, to study the characteristics of each subject, a body composition analyzer (InBody S10, Biospace Co., Ltd., Chiyoda-ward, Tokyo, Japan) that uses bioelectric impedance was used to measure the subjects' fat and muscle masses.

### 2.4. Analysis Methods

To determine the intensity of physical activity of the SSEs, expired gas analysis indices were separated into the following groups: resting, Session 1, and Sessions 2–6. Means ± standard deviation were calculated for each group. The normality of the continuous data was checked using the Shapiro-Wilk tests. A one-way analysis of variance (ANOVA) for repeated measures was used to compare the cardiorespiratory responses (Bonferroni's multiple comparison test was used as post hoc analysis). Subsequently, the groups were divided by sex and age to examine whether there were differences among the indices. Between groups, differences in mean values were tested for statistical differences with unpaired *t*-tests and Mann-Whitney tests, when appropriate. SPSS Version 20.0 was used for statistical analysis (IBM SPSS, Tokyo, Japan). Statistical significance was set at 5%.

## 3. Results

The basic characteristics of the subjects are shown in Table 2. The average age was 75.2 years for men and 73.6 years for women. The average height was 162.0 cm for men and 148.4 cm for women. The average weight was 60.8 kg for men and 49.8 kg for women. The average body mass index (BMI) was 23.0 kg/m^2^ for men and 22.5 kg/m^2^ for women. There were no subjects with a BMI <18.5 kg/m^2^, which is considered to be “underweight.” As for subjects with a BMI ≥25.0 kg/m^2^, there was one man and one woman with BMI values of 25.3 kg/m^2^ and 25.8 kg/m^2^, respectively. The average resting heart rate was 65.6 bpm for men and 71.7 bpm for women. Anomalous beat was not observed during the test. Resting systolic blood pressure was 147.2 mmHg for men and 145.7 mmHg for women. There were 4 men and 4 women whose resting systolic blood pressure was ≥140 mmHg. Resting diastolic blood pressure was 86.0 mmHg for men and 79.7 mmHg for women. There were 3 men and 2 women whose resting diastolic blood pressure was ≥90 mmHg.

Cardiorespiratory and energy expenditure responses of the 3 groups (resting, Session 1, and Sessions 2–6) are shown in Table 3. The average heart rates at resting, Session 1, and Sessions 2–6 were 68.6 ± 9.8 bpm, 80.9 ± 11.2 bpm, and 84.8 ± 12.0 bpm, respectively. Oxygen pulse (O_2_ pulse) indicates the amount of oxygen consumed after every heartbeat. The average O_2_ pulse values at resting, Session 1, and Sessions 2–6 were 3.8 ± 1.0 mL, 5.2 ± 1.4 mL, and 5.9 ± 1.5 mL, respectively. Minute ventilation (*V*_*E*_) refers to the amount of ventilation in 1 minute. The average *V*_*E*_ values at resting, Session 1, and Sessions 2–6 were 9.1 ± 1.7 mL, 14.1 ± 2.7 mL, and 17.0 ± 2.5 mL, respectively. The *V*_O_2__, HR, and *V*_*E*_ showed significant increases in the SSEs. The average METs of the subjects were 1.6 ± 0.3 during Session 1 and 1.9 ± 0.3 during Sessions 2–6.

The cardiorespiratory response during the SSEs and the implementation conditions by sex and age are described below (data not shown). When comparing by sex, METs were significantly higher (*P* = 0.022) for men than for women while performing Session 1 of the SSEs. However, there was no significant difference (*P* = 0.109) in METs for the overall SSEs. Furthermore, there were no significant differences in HR, heart rate reserve (HRR), modified Borg's scale, time required to perform the SSEs, and number of stick droppings on the floor. When comparing by age groups, there was a significant difference (*P* = 0.001) in the increase in HR during Session 1 between the early elderly (between 60 and <74 years) and the late elderly (≥75 years). However, there was no significant difference (*P* = 0.404) in the overall SSEs. Subsequently, HRR during Session 1 was also significantly higher (*P* = 0.008) in the early elderly than in the late elderly, but there was no significant difference (*P* = 0.407) for the overall SSEs. As for the number of unintentional stick droppings, the late elderly dropped the sticks significantly more often (*P* = 0.020) than the early elderly individuals. There were no significant differences in METs, RPE, and time required to perform the exercises.

## 4. Discussion

Upon reviewing the data for height, weight, and resting blood pressure of the study subjects, they were found to be a representative sample of the elderly population in Japan [12]. The METs for intensity of the SSEs were 1.6 ± 0.3 for Session 1 (warming up) and 1.9 ± 0.3 for Sessions 2–6. The physical activity of the SSEs was equivalent to the values stated by Ainsworth et al. [13], such as sitting and talking in person (1.5 METs), fidgeting while standing (1.8 METs), and walking during household work (2.0 METs). For all the samples, the HRR (%) during the SSEs was 15.8 ± 7.8, 21.0 ± 10.4, and 20.0 ± 9.6 for Session 1, Sessions 2–6, and the overall exercises, respectively (data not shown). Taking into consideration the cardiorespiratory responses of these exercises, the intensity level of the SSEs would be categorized as a sedentary to light intensity activity [14]. Walking, which is often brought up as a physical activity intervention for the elderly, is 3.5 METs (walking at a moderate pace) [13]. Similarly, Tai Chi is reported to be 4 METs [15]. Compared to these exercises, the SSEs' intensity level is quite low. The SSEs have been found to significantly improve physical functions, to maintain cognitive function, and to reduce the number of fallers [9]. In addition, although the SSE is the exercises of very low levels, it proved nearly as effective as Tai Chi [9]. The American College of Sports Medicine recommends that late elderly people (75 years and above) and elderly people with movement limitations should start with an exercise program with an intensity of 3 METs or below that has a low risk [7]. The results of this study show that it is possible to adapt the SSEs for late elderly people (75 years and above) and elderly people with decreased physical fitness.

Ample studies have demonstrated that high-intensity exercise interventions seem to be more effective in improving physical functioning than low-intensity exercise interventions [16, 17], whereas a few studies have reported that low-intensity exercise could improve physical performance in the elderly [18, 19]. However, it is unclear whether low-intensity exercise training can increase physical functioning. In addition, little is known about how the SSEs contribute to the maintenance improvement of physical performance in the elderly. Therefore, further studies are needed in order to better understand the possible effects of the SSEs.

Reports have shown that negative emotions arise when exercising above the ventilatory threshold (*V*_*T*_) [20, 21], and excessive exercise intensity may affect adherence [22–24]. Based on the changes in carbon dioxide output (*V*_CO_2__) and HR during the SSEs, it appears that *V*_*T*_ was not exceeded in this study. In our previous study, adherence to the SSEs was high (87.8%) [9]. This may be attributed to the low-intensity level of the exercises. However, according to the subjective RPE of the physical activity intensity level, the median was 4.0, and the scores, 1.0 (very weak) to 5.0 (strong), were widely distributed between the 25th and 75th percentiles (data not shown). Since all of the subjects from this study had no previous experience with the SSEs, they may have gotten confused with difficulty in performing the exercises and the exercise intensity. When a comparison was made by sex, the men's METs in Session 1 were significantly higher than the women's. Considering that there was no significant difference between men and women in the increased HR and HRR, there may have been bias. In the future, further examinations with an increased sample size are needed. When a comparison was made by age, the increase in HR from resting to Session 1 of the SSEs was significantly smaller in the late elderly than in early elderly individuals. Furthermore, the HRR of Session 1 was significantly lower (*P* = 0.056) in the late elderly than in the early elderly, which was the same trend observed in the METs category. These results indicate how low the energy expenditure response was for the late elderly in Session 1. A study reported that there is a delay in the circulatory response in elderly individuals at the beginning of exercise [25, 26]. The reason behind this delay may be decreased vagus nerve activity due to aging and a delayed *β* adrenergic response [27]. Ellestad [28] and Kligfield and Lauer [29] reported that failure to increase HR appropriately during physical activity is linked to cardiovascular diseases and may increase the death rate. When the late elderly engage in Session 1, we may need to monitor the changes in HR and check whether they report any subjective symptoms. In addition, when the frequency of stick dropping was compared by age, the late elderly dropped their sticks significantly more often than the early elderly individuals. While the action of picking up the stick is an effective movement and a good opportunity to practice shifting weight at the same time, we may need to be vigilant about the psychological effects caused by the accumulation of failed attempts.

## 5. Conclusion

Based on cardiorespiratory and energy expenditure responses, it was found that the physical activity intensity of the SSEs performed by elderly individuals corresponds to a sedentary to light physical activity. Since the SSEs are lighter in intensity than other exercises such as walking, we believe the SSEs may be adapted for late elderly individuals and other elderly people with decreased physical fitness. However, since late elderly individuals have difficulty in elevating their HR at the start of the exercises (Session 1) and since there is an increase in stick droppings, we must be vigilant while they perform the SSEs.

## Figures and Tables

**Table 1:** Content, times, and duration of 6 steps of short stick exercises for elderly people.

Step number	Contents		Times
1	Warming-up exercise		5 times each

2	Throwing and catching stick		3 times each
	One-hand version	Throwing and catching stick	
		Catching stick with palm down	
		Catching stick vertically	
		Clapping hands before catching	

3	Throwing and catching stick		3 times each
	Both-hands version	Trying to throw and catch	
		Catching stick with palms down	
		Catching stick vertically	
		Catching stick with one palm up and the other palm down	

4	Keeping stick balanced		3 times each
		Keeping stick balanced on palm	
		Keeping balance of stick on floor	
		Keeping balance of stick on floor and clapping hands	
		Putting stick on back of your hand	

5	Flipping stick over		3 times each
		Flipping stick toward you and catching	
		Flipping stick forward and catching	

6	Dropping the stick		3 times each
		Dropping and catching stick	
		Dropping stick and catching stick between knees	

**Table 2:** Characteristics of subjects.

	Male	Female
	(*n* = 10)	(*n* = 10)
Age (yrs)	75.2 ± 9.8	73.6 ± 8.3
Body height (cm)	162.0 ± 4.0	148.4 ± 6.7
Body weight (kg)	60.8 ± 6.8	49.8 ± 6.3
BMI (kg/m^2^)	23.0 ± 2.7	22.5 ± 2.1
Fat mass (kg)	15.9 ± 5.0	16.4 ± 3.6
Muscle mass (kg)	42.1 ± 4.4	31.4 ± 4.2
Percent body fat (%)	25.9 ± 6.2	32.8 ± 5.7
Muscle mass/body weight (%)	69.4 ± 6.7	63.1 ± 5.5
Resting HR (bpm)	65.6 ± 10.2	71.7 ± 8.8
Resting systolic BP (mmHg)	147.2 ± 22.2	145.7 ± 23.0
Resting diastolic BP (mmHg)	86.0 ± 9.0	79.7 ± 10.1

Data are listed as mean ± SD.

BMI: body mass index.

HR: heart rate.

BP: blood pressure.

**Table 3:** Cardiorespiratory and energy expenditure responses of subjects during short stick exercises.

	Resting (R)	Session 1 (S1)	Sessions 2–5 (S2–5)	ANOVA^∗1^	Multiple comparisons^∗2^		
					R versus S1	S1 versus S2–5	R versus S2–5
*V*_O_2__ (mL/min)	258.8 ± 57.9	422.9 ± 110.9	491.5 ± 102.6	<0.001	<0.001	<0.001	<0.001
HR (bpm)	68.6 ± 9.8	80.9 ± 11.2	84.8 ± 12.0	<0.001	<0.001	0.018	<0.001
O_2_ pulse (mL)	3.8 ± 1.0	5.2 ± 1.4	5.9 ± 1.5	<0.001	<0.001	<0.001	<0.001
*V*_*E*_ (mL/min)	9.1 ± 1.7	14.1 ± 2.7	17.0 ± 2.5	<0.001	<0.001	<0.001	<0.001
METs	—	1.6 ± 0.3	1.9 ± 0.3	—	—	—	—

Data are listed as mean ± SD, and other data are listed as *P* value.

Session 1: warming up.

Sessions 2–5: throwing, catching, keeping, flipping, and dropping a short stick.

*V*_O_2__: oxygen uptake, HR: heart rate, O_2_ pulse: oxygen pulse, *V*_*E*_: minute ventilation, METs: metabolic equivalents.

^∗1^Repeated one-way ANOVA, ^∗2^Bonferroni's multiple comparisons.
